# Selective κ Opioid Antagonists nor-BNI, GNTI and JDTic Have Low Affinities for Non-Opioid Receptors and Transporters

**DOI:** 10.1371/journal.pone.0070701

**Published:** 2013-08-14

**Authors:** Thomas A. Munro, Xi-Ping Huang, Carmela Inglese, Maria Grazia Perrone, Ashlee Van't Veer, F. Ivy Carroll, Cécile Béguin, William A. Carlezon, Nicola A. Colabufo, Bruce M. Cohen, Bryan L. Roth

**Affiliations:** 1 McLean Hospital, Belmont, Massachusetts, United States of America; 2 Department of Psychiatry, Harvard Medical School, Boston, Massachusetts, United States of America; 3 School of Chemistry and Bio21 Institute, University of Melbourne, Parkville, Australia; 4 National Institute of Mental Health Psychoactive Drug Screening Program and Department of Pharmacology, University of North Carolina School of Medicine, Chapel Hill, North Carolina, United States of America; 5 Dipartimento di Farmacia-Scienze del Farmaco, Università degli Studi di Bari, Bari, Italy; 6 Center for Organic and Medicinal Chemistry, Research Triangle Institute, Research Triangle Park, North Carolina, United States of America; University of North Dakota, United States of America

## Abstract

**Background:**

Nor-BNI, GNTI and JDTic induce selective κ opioid antagonism that is delayed and extremely prolonged, but some other effects are of rapid onset and brief duration. The transient effects of these compounds differ, suggesting that some of them may be mediated by other targets.

**Results:**

In binding assays, the three antagonists showed no detectable affinity (*K*_i_≥10 µM) for most non-opioid receptors and transporters (26 of 43 tested). There was no non-opioid target for which all three compounds shared detectable affinity, or for which any two shared sub-micromolar affinity. All three compounds showed low nanomolar affinity for κ opioid receptors, with moderate selectivity over μ and δ (3 to 44-fold). Nor-BNI bound weakly to the α_2C_-adrenoceptor (*K*_i_ = 630 nM). GNTI enhanced calcium mobilization by noradrenaline at the α_1A_-adrenoceptor (EC_50_ = 41 nM), but did not activate the receptor, displace radioligands, or enhance PI hydrolysis. This suggests that it is a functionally-selective allosteric enhancer. GNTI was also a weak M_1_ receptor antagonist (*K*_B_ = 3.7 µM). JDTic bound to the noradrenaline transporter (*K*_i_ = 54 nM), but only weakly inhibited transport (IC_50_ = 1.1 µM). JDTic also bound to the opioid-like receptor NOP (*K*_i_ = 12 nM), but gave little antagonism even at 30 µM. All three compounds exhibited rapid permeation and active efflux across Caco-2 cell monolayers.

**Conclusions:**

Across 43 non-opioid CNS targets, only GNTI exhibited a potent functional effect (allosteric enhancement of α_1A_-adrenoceptors). This may contribute to GNTI's severe transient effects. Plasma concentrations of nor-BNI and GNTI may be high enough to affect some peripheral non-opioid targets. Nonetheless, κ opioid antagonism persists for weeks or months after these transient effects dissipate. With an adequate pre-administration interval, our results therefore strengthen the evidence that nor-BNI, GNTI and JDTic are highly selective κ opioid antagonists.

## Introduction

**Figure 1:**
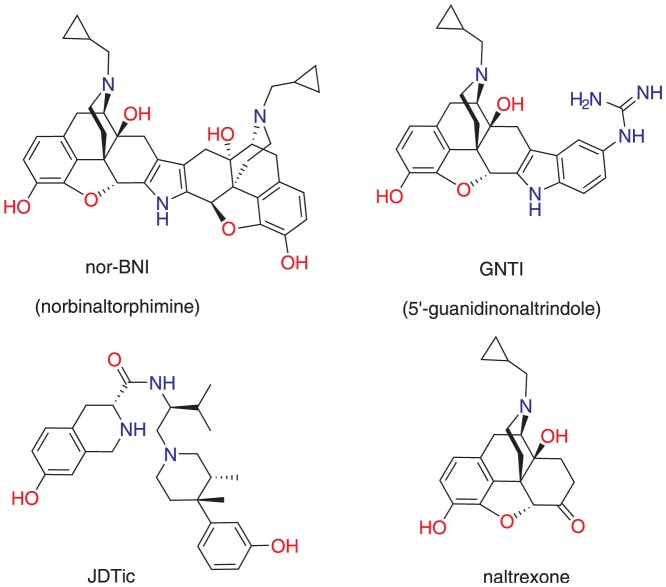
Structures of opioid antagonists nor-BNI, GNTI, JDTic and naltrexone.

Selective κ (kappa) opioid antagonists may have therapeutic potential against conditions such as depression and anxiety disorders [1], [2]. The best-established agents in this class, shown in Figure 1, are nor-BNI, GNTI and JDTic. *In vitro*, these compounds are potent and selective antagonists at the κ opioid receptor (κ-OR), with much lower potency at μ- (mu) and δ- (delta) OR [3]. They are therefore generally considered κ-selective, but little evidence is available on binding and activity at other receptors, ion channels, transporters and enzymes.

### Delayed, prolonged κ opioid antagonism

The pharmacodynamics of these compounds *in vivo* differ dramatically from other opioid antagonists. Attainment of maximal κ opioid antagonism may be delayed by hours or days, compared to minutes for competitive antagonists like naloxone [4]. Duration of action is also extremely long; while competitive antagonists are typically effective for only hours or at most days, κ antagonism can persist for weeks or months after nor-BNI, GNTI or JDTic [4]. To account for this abnormal timecourse, it was long presumed that these compounds were slowly absorbed and eliminated. Recently, studies have suggested instead that nor-BNI, GNTI and JDTic activate the enzyme c-Jun N-terminal kinase 1 (JNK1, MAPK8), causing desensitization of κ-OR that persists long after the compounds are eliminated [5]. Thus, these compounds appear to induce functional antagonism via a non-competitive mechanism. Short-acting κ antagonists did not activate JNK1 [6].

### Transient effects

Surprisingly, despite the extremely protracted timecourse of κ antagonism, other effects of nor-BNI, GNTI and JDTic are of rapid onset and brief duration [4], [7]. After subcutaneous (s.c.) administration to mice, nor-BNI and GNTI induce scratching that is maximal within 20 minutes and lasts less than two hours [8], [9]. Nor-BNI and JDTic inhibit self-administration of ethanol by rats at 2 hours, but not 24 hours [10]. Nor-BNI also reduces the maximal responding rate to intracranial self-stimulation in rats over the first two hours, but not after 24 hours [11]. In mice, GNTI strongly inhibits locomotor activity within 20 minutes, but the effect dissipates within three hours [12]. Nor-BNI inhibits locomotor activity in rats on the day of administration, but not the next day [13]. Despite its high κ-selectivity *in vitro* [3], nor-BNI produces transient μ and δ antagonism in mice, again with rapid onset and lasting only a few hours [14], [15]. Thus, all three of these compounds have transient effects.

### Potential mechanisms of transient effects

These effects are maximal when κ antagonism is submaximal (<2 hours), and undetectable when antagonism is maximal (24 hours) [4], [16]. This strongly suggests that these effects are not mediated by antagonism of the endogenous κ opioid dynorphin A (dynA). We recently found that nor-BNI, GNTI and JDTic are rapidly absorbed and eliminated from plasma after intraperitoneal (i.p.) administration, coinciding closely with the timecourse of their transient effects [7]. We have argued that such effects are likely to result from competitive, reversible mechanisms, while the delayed, prolonged timecourse of κ antagonism is more consistent with a non-competitive, irreversible process such as desensitization [7].

While κ antagonism cannot plausibly account for these transient effects, the fact that some of them are caused by several of these compounds suggests that they may nonetheless be κ-OR-mediated. They might, for instance, result from inverse or biased agonism. However, effects seen after only one of these agents suggest the involvement of targets other than κ opioid receptors (κ-OR). Dramatically, we found that administration of a high dose of GNTI to mice (30 mg/kg i.p.) caused ataxia, convulsions and death within 18 minutes. By contrast, even at 100 mg/kg, nor-BNI and JDTic caused no convulsions or deaths [7]. Naltrexone is also much less toxic to mice (LD_50_ = 570 mg/kg s.c.) [17]. The GNTI analogue 5′-aminomethylnaltrindole was also recently reported to induce gasping and convulsions after a high dose (20 mg/kg i.p.), suggesting that this series of compounds may act on a common target other than κ-OR [18].

Nor-BNI [19], GNTI [20] and JDTic [21] all inhibit deprivation-induced feeding in rats. Surprisingly, however, while nor-BNI also inhibits feeding induced by butorphanol or neuropeptide Y, GNTI does not [20]. These contrasting profiles suggest that nor-BNI or GNTI, or both, interact with another target that modulates feeding. Evidence has recently been presented that GNTI-induced scratching is mediated by targets other than κ-OR [9], [22]–[25]. In addition to the discrepancy in timecourse noted above, scratching was not inhibited by naloxone, and was equally intense in κ-OR knockout mice [9]. Screening of 1 µM GNTI against a panel of 34 non-opioid receptors and ion channels revealed substantial binding only to M_1_ muscarinic receptors (M_1_-R) [9]. *In vivo*, M_1_ agonist McN-A-343 inhibited GNTI-induced scratching, consistent with the *in vitro* evidence for the involvement of this receptor [9], [25].

Less evidence is available on the affinities of nor-BNI and JDTic for non-opioid targets. Nor-BNI has been reported to show very low affinity (*K*_i_>1 µM) for the *N*-methyl-d-aspartate (NMDA) receptor [26], and to weakly modulate dynA binding to the same receptor [27]. No binding was detected at 10 µM to the glucocorticoid receptor NR3C1 [28]. Nor-BNI's affinity for the opioid-like nociceptin/orphanin FQ receptor (NOP) is also negligible; one study found low affinity (*K*_i_ = 780 nM) [29], while another detected no binding at 10 µM [30].

To summarize, the mechanisms underlying the transient effects of nor-BNI, GNTI and JDTic are unknown, and differences between these compounds suggest that non-opioid targets may be involved. In this study, we sought evidence of such interactions. Using the resources of the National Institute of Mental Health's Psychoactive Drug Screening Program [31], we measured the binding affinities of nor-BNI, GNTI and JDTic for 46 receptors, ion channels and neurotransmitter transporters, and evaluated hits in functional assays. We also tested for active efflux from Caco-2 cell monolayers, which express multiple efflux transporters.

## Results

### Nor-BNI, GNTI and JDTic bind selectively to opioid receptors

As expected, nor-BNI, GNTI and JDTic bound selectively to κ- over μ- and δ-OR, albeit with lower selectivity (3 to 44-fold) than in some previous reports [3]. All three compounds had very low affinities for non-opioid targets generally (Table 1): at most targets, (26 of 43), none of the three compounds bound detectably at 10 µM. Furthermore, there was no non-opioid target to which all three compounds bound detectably at 10 µM, or for which any two shared sub-micromolar affinity. Only three sub-micromolar affinities were detected: nor-BNI for the α_2C_-adrenoceptor (α_2C_-AR, K_i_ = 630 nM), and JDTic for the opioid-like receptor NOP (K_i_ = 12 nM) and the noradrenaline (norepinephrine) transporter (NET, K_i_ = 54 nM).

**Table 1:** Binding affinities of nor-BNI, GNTI and JDTic for 46 neurotransmitter receptors and transporters, determined by radioligand displacement.

	nor-BNI	GNTI	JDTic		nor-BNI	GNTI	JDTic
Target	*K*_i_ (nM)			Target	*K*_i_ (nM)		
**κ**	**4**	**3**	**1**	**β_3_**			
**μ**	**41**	**54**	**3**	**D_1_**			
**δ**	**20**	**58**	**44**	D_2_			
**NOP**	4,400	2,500	**12**	**D_3_**			
**5-HT_1a_**	1,100			**D_4_**			
**5-HT_1b_**				**D_5_**	3,900	8,200	
**5-HT_1d_**	1,100			**DAT**			4,400
**5-HT_1e_**				**GABA_A_**			
**5-HT_2a_**				**GABA_A_ (BZP)**			
**5-HT_2b_**				**H_1_**			
**5-HT_2c_**				**H_2_**			1,800
**5-HT_3_**				**H_3_**			
**5-HT_5a_**				**H_4_**			
**5-HT_6_**				**M_1_**			
**5-HT_7_**				**M_2_**		5,400	
**α_1D_**				**M_3_**			
**α_1A_**		9,800		**M_4_**			
**α_1B_**		9,500	4,800	**M_5_**			
**α_2A_**	4,900			**NET**			**54**
**α_2B_**	1,500			**NMDA**			
**α_2C_**	**630**	2,200		**SERT**			
**β_1_**				**σ_1_**			1,900
**β_2_**				**σ_2_**			

Submicromolar affinities are shown in bold; blank cells indicate *K*_i_≥10 µM. For details (uncertainty, radioligand, membrane type, species), see Table S1. For binding curves, see File S1.

### Nor-BNI does not bind to NMDA receptors

Contrary to previous reports, we detected no binding by nor-BNI to NMDA receptors. However, this receptor's multiple binding sites complicate comparisons. Nor-BNI was reported to weakly displace ^3^H]CGP-39,653 from the glutamate site [26], and to modulate displacement of [^3^H]-MDL-105,519 from the glycine site by dynA [27]. In our hands, nor-BNI did not displace [^3^H]MK-801 from the phencyclidine (cation channel) site at 10 µM,. This does not exclude the possibility of binding to the glutamate and glycine sites. Note that in previous reports, binding to these sites was only detectable at nor-BNI concentrations above 1 µM, consistent with the negligible affinity observed here.

### GNTI is an allosteric enhancer of α_1A_-AR (EC_50_ = 41 nM)

In initial tests, GNTI showed affinity for α_1A_ adrenoceptors (α_1A_-AR). We therefore measured functional activity in an intracellular calcium mobilization assay. Surprisingly, even at 30 µM GNTI neither activated α_1A_-AR nor inhibited activation by noradrenaline (norepinephrine). To reconcile this apparent discrepancy, we tested for allosteric enhancement of noradrenaline's effects.

**Figure 2:**
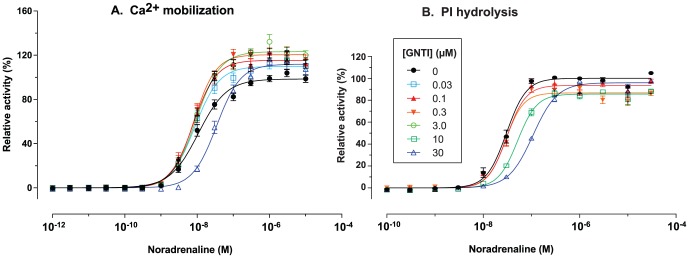
GNTI enhances maximal Ca^2+^ mobilization by noradrenaline at α_1A_-AR without affecting potency (A); maximal PI hydrolysis is not increased (B). Some intermediate curves have been omitted for clarity. Error bars represent mean ± S.E.M. For raw data, see Datasets S1 and S2.

In the calcium mobilization assay, GNTI did not activate α_1A_-AR, but increased the maximal response to noradrenaline by up to 20% without affecting potency (Figure 2A). This suggests that GNTI is an allosteric enhancer, or positive allosteric modulator. The EC_50_ of GNTI for this enhancement was 41 nM (95% CI = 24 to 72 nM). At the highest concentration of GNTI tested (30 µM), the potency of noradrenaline and the increase in maximal response were both reduced, suggesting that weak competitive antagonism occurs at this concentration.

By contrast, GNTI slightly reduced the maximal response to noradrenaline in an assay measuring hydrolysis of phosphatidylinositol 4,5-bisphosphate (PI); weak competitive antagonism again occurred above 10 µM (Figure 2B). This discrepancy was surprising, since PI hydrolysis leads to calcium mobilization. However, other pathways governing α_1A_-AR-mediated calcium mobilization reportedly exist [32]. While fluorescent dye-based calcium assays of the kind we used can generate artefacts [33], the lack of response to 30 µM GNTI in the absence of noradrenaline argues against this (Figure 2A). These results suggest that GNTI imposes functional selectivity on noradrenaline's actions at α_1A_-AR, selectively enhancing efficacy towards a PI-independent signalling pathway.

Upon retesting, we were unable to replicate our initial finding of high binding affinity: GNTI displaced [^125^I]HEAT from α_1A_-AR only at concentrations approaching 10 µM (Table 1). In addition, 10 µM GNTI did not detectably modulate the displacement of [^125^I]HEAT by noradrenaline. This is not inconsistent with the functional results, since allosteric modulators can affect efficacy independently of affinity [34]. However, it is fortuitous that the apparently incorrect initial result led us to investigate further.

To summarize, the above data indicate that GNTI acts as a functionally-selective allosteric enhancer of α_1A_-ARs. Under these conditions, GNTI increases maximal calcium mobilization by noradrenaline, but does not enhance PI hydrolysis.

### GNTI is a weak M_1_ antagonist (*K*_B_ = 3.7 µM)

**Figure 3:**
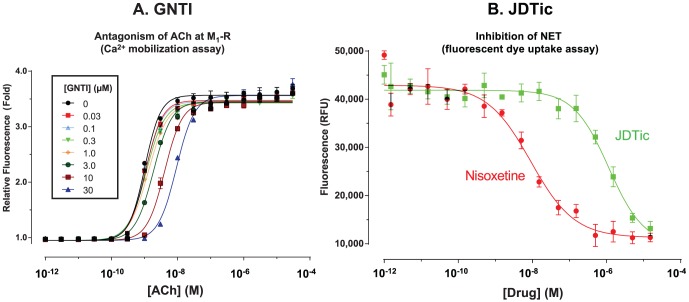
GNTI is a weak antagonist of acetylcholine at M_1_-R (A); JDTic weakly inhibits the noradrenaline transporter (B). Error bars represent mean ± S.E.M. For raw data, see Datasets S3 and S4.

GNTI showed negligible affinity for all non-opioid targets (*K*_i_>2 µM). In a previous report [9], GNTI gave 52% displacement from M_1_-R at 1 µM, while we observed only 49% displacement at 10 µM. One potential reason for this small difference is our use of a different radioligand, [^3^H]3-quinuclidinyl benzilate (QNB); the previous report used [^3^H]*N*-methylscopolamine. Given the convergent evidence reported previously for the involvement of M_1_-R [9], we nonetheless tested for functional activity using an intracellular calcium mobilization assay (Figure 3A). GNTI did not activate M_1_-R, and inhibited the effect of acetylcholine with very low potency: *K*_B_ = 3.7 µM (95% CI = 3.2 to 4.3 µM). Given the consistently sub-nanomolar potency of GNTI at κ-OR in functional assays [3], these results confirm >3,000-fold selectivity.

### JDTic weakly inhibits noradrenaline transport (IC_50_ = 1.1 µM)

JDTic bound to the noradrenaline transporter with moderately high affinity (*K*_i_ = 54 nM versus [^3^H]nisoxetine). However, in a functional assay (Figure 3B), inhibition of transport only occurred at much higher concentrations (IC_50_ = 1.1 µM, 95% CI = 0.5 to 2.6 µM). This was not due to assay insensitivity, since the positive control nisoxetine exhibited high potency (IC_50_ = 9 nM, 95% CI = 5 to 15 nM). The surprisingly low potency of JDTic suggests that it may bind with high affinity to an allosteric site, modulating the affinity of [^3^H]nisoxetine without affecting noradrenaline transport. Evidence for at least one affinity-modulating allosteric site in NET has been reported previously [35]. The inhibition seen at high concentrations may be due to JDTic binding with lower affinity to a transport-modulating site.

### JDTic weakly inhibits NOP (pA_2_∼7.1)

**Figure 4:**
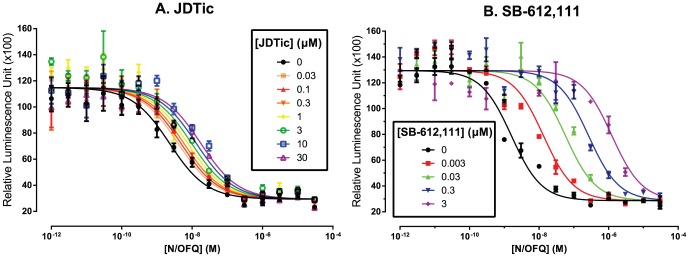
Antagonism of N/OFQ at NOP by JDTic (A) and SB-612,111 (B): inhibition of cAMP production. Error bars represent mean ± S.E.M. For raw data, see Dataset S5.

JDTic bound with high affinity to NOP (*K*_i_ = 12 nM, 95% CI = 7 to 20; Table 1), displacing the endogenous agonist nociceptin/orphanin FQ ([^3^H]N/OFQ). Nor-BNI showed negligible affinity, consistent with prior reports [29], [30], as did GNTI. Surprisingly, despite its high affinity and lack of efficacy, JDTic only weakly inhibited the response to N/OFQ in a functional assay (inhibition of cyclic AMP production: pA_2_ = 7.1, 95% CI = 5.9 to 8.3, Figure 4A). This would be equivalent to a *K*_B_ of 73 nM for a competitive antagonist (95% CI = 4.6 to 1,200 nM). Maximally-effective concentrations of N/OFQ (>100 nM) were not affected even by extremely high concentrations of JDTic (30 µM); this saturable effect suggests negative allosteric modulation rather than competitive antagonism (Figure 4A). The full displacement of [^3^H]N/OFQ we observed in the binding assay is therefore surprising. Note, however, the low concentration of [^3^H]N/OFQ used: 0.74 nM, equal to the *K*_d_. At this concentration of N/OFQ, JDTic almost abolished response in the functional assay (Figure 4A). The positive control SB-612,111 caused potent surmountable antagonism, abolishing the response to maximally-effective concentrations of N/OFQ (pA_2_ = 9.8, 95% CI = 9.4 to 10.2, Figure 4B). This is concordant with a prior report [36]. The extreme potency of JDTic in κ-OR functional assays (*K*_e_<20 pM) [3] thus confers at least >1,000-fold selectivity over NOP and NET, and the incomplete NOP antagonism we observed suggests that effective selectivity is greater still.

### Nor-BNI, GNTI and JDTic undergo active efflux from Caco-2 cells

We next evaluated membrane permeation and active efflux, measuring permeation through Caco-2 cell monolayers by UV spectroscopy. Nor-BNI, GNTI, JDTic and naltrexone all appeared to undergo active efflux, as indicated by an efflux ratio greater than 1 (Table 2). Note, however, that compounds with efflux ratios in this moderate range (<18) may be modulators rather than substrates [37]. Contrary to these results, in previous tests using Caco-2 and other cell lines, naltrexone was not found to undergo active efflux [7], [38], [39]. The permeabilities we observed for all four compounds were also orders of magnitude higher than we previously observed in LLC-PK1 cell layers [7]. We are unaware of the reason(s) for these discrepancies; Caco-2 cell populations exhibit substantial heterogeneity, with differences in the expression of particular transporters and high inter-laboratory variation in permeation rates [40], [41]. Nonetheless, the finding that nor-BNI, GNTI and JDTic are subject to active efflux is consistent with the low brain uptake we observed previously [7]. We found there that nor-BNI and GNTI were not substrates of human permeability glycoprotein (P-gp); taken together our results suggest that these antagonists may be substrates of another efflux transporter.

**Table 2:** Mean permeation rates and efflux ratios in Caco-2 cell monolayers.

	P_app_		Efflux ratio
Compound	B→A	A→B	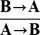
	nm/s		
**Nor-BNI**	3,100	510	6.1
**GNTI**	2,700	880	3.1
**JDTic**	3,100	600	5.2
**Naltrexone**	2,700	590	5.7

Data are the mean of three independent determinations (samples in triplicate) each with SEM <10%. A: apical; B: basolateral.

## Discussion

### Potential consequences of α_1A_-AR modulation by GNTI

**Figure 5:**
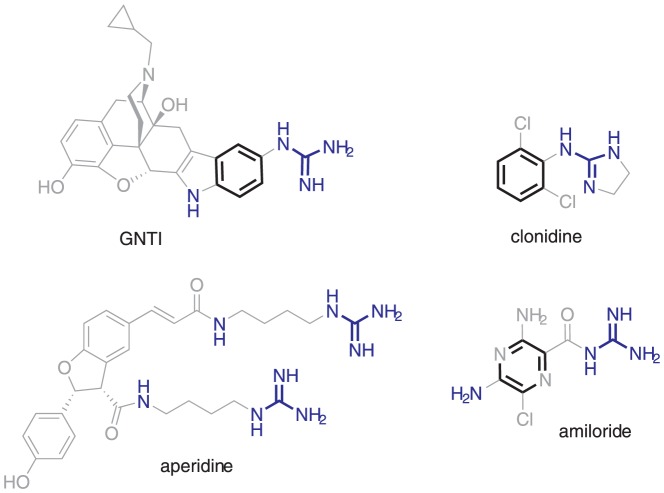
Structural similarities between GNTI and α_1_-AR ligands.

Our results indicate that GNTI is a moderately potent allosteric enhancer of α_1A_-AR. It is interesting to note that GNTI is structurally similar to other α_1_-AR ligands (Figure 5). Aperidine and analogues act as α_1A_-AR antagonists, and a guanidine substituent is required for activity [42]. The peptide ρ-TIA (not shown) is a negative allosteric modulator of α_1_-AR, also featuring a guanidine substituent that is essential for activity [43].

Clonidine is an allosteric modulator of α_1A_-AR [44] featuring a phenylimidazoline moiety similar to the phenylguanidine group of GNTI (Figure 5). Clonidine and many related ligands appear to bind in an allosteric pocket of the α-AR, unlike the orthosteric phenethylamines [45]. The structurally-related sodium channel blocker amiloride and analogues (Figure 5) are negative allosteric modulators of α_1A_-AR [46]. These prior results indicate that α_1A_-AR possesses an allosteric site that binds structurally diverse guanidine-substituted ligands and related compounds. Interestingly, it has recently been reported that unlike the orthosteric agonist noradrenaline, the imidazolines are functionally selective at α_1A_-AR, with a bias towards calcium mobilization [47].

Allosteric enhancement of noradrenaline's actions at α_1A_-AR may plausibly contribute to some of GNTI's transient effects. For instance, α_1_ agonists have been reported to inhibit feeding [48] and serotonin-induced itch [49]. The ataxia and death we observed after high-dose GNTI [7] may also be related, since α_1_-AR agonists have been found to promote catalepsy [50] and to raise blood pressure [51]. Furthermore, GNTI's potency as an α_1A_ enhancer (41 nM) is comparable to our estimate of the unbound brain concentration after a high dose of GNTI (5 nM after 39 mg/kg) [7]. Thus, it is plausible that synergy between this target and κ-OR might contribute to centrally-mediated effects of GNTI such as inhibition of feeding and locomotion. Such an interaction is less plausible for M_1_-R, towards which the potency of GNTI is orders of magnitude lower.

### Do M_1_ receptors contribute to GNTI-induced scratching? Remaining questions

Our finding that GNTI acts as a weak M_1_ antagonist is consistent with a previous report of weak binding to that receptor [9]. Nonetheless, it is unclear whether the extremely low potency we observed would be relevant *in vivo*. In the prior report, an M_1_ agonist (McN-A-343) inhibited GNTI-induced scratching in mice, providing apparent *in vivo* confirmation that GNTI acts upon this receptor [9], [25]. An M_1_ antagonist had no effect. However, those results are difficult to interpret, for several reasons. Firstly, in earlier reports M_1_ agonists induced scratching, while antagonists inhibited it [52]. Thus, the reported inhibition of GNTI-induced scratching by an M_1_ agonist is paradoxical. Secondly, McN-A-343 was administered intrathecally (i.t.), while GNTI was injected s.c. [9]. Due to GNTI's low potency and extremely low central uptake [7], this would be unlikely to result in a detectable effect on spinal M_1_-R. Indeed, GNTI induces scratching after s.c. but not i.t. administration [9], while M_1_ agonists show the opposite profile [53], suggesting that any interaction would be indirect. Finally, McN-A-343 is poorly selective for M_1_ receptors [54], so the involvement of other targets cannot be ruled out. Collectively, this evidence is more consistent with an indirect, downstream interaction than a competitive one between GNTI and McN-A-343 at spinal M_1_ receptors. Further exploration of this issue would benefit from the use of more selective M_1_ ligands, administered by the same route as GNTI.

### Potential roles of peripheral non-opioid targets in the transient effects of nor-BNI and GNTI

It remains plausible that peripheral M_1_ receptors may be involved in some transient effects of GNTI. We previously observed peak unbound GNTI concentrations in plasma of 2 µM at a dose of 10 mg/kg, and 8 µM at 39 mg/kg [7]. These concentrations would be expected to result in some peripheral M_1_ antagonism, given *K*_B_ = 3.7 µM. At these concentrations, detectable receptor occupancy would also be expected at five other peripheral targets, including M_2_-R (Table 1). Similarly, the peak unbound concentration of nor-BNI in plasma was 3 µM at 10 mg/kg [7], comparable to the affinities determined here for six non-opioid targets. Thus, due to the very low uptake of nor-BNI and GNTI, effective levels in brain require very high plasma concentrations after peripheral administration. These concentrations will result in transient occupancy of peripheral targets for which these compounds have low affinity, reducing their effective selectivity. The relevance of this *in vivo* is unclear. While it seems plausible that peripheral receptors might influence responses such as scratching, this seems less likely for behaviours such as locomotion and feeding. The peak unbound concentration of JDTic in plasma was 100 nM after a 10 mg/kg dose [7]. Based on our results, this concentration would be expected to yield substantial binding to NOP and NET, but little or no functional effect. Moreover, this concentration would not be expected to produce substantial occupancy of any of the other non-opioid targets studied here.

Plasma concentrations of nor-BNI, GNTI and JDTic decline by over 80% within 4 hours, and over 98% within 24 hours [7]. By contrast, κ opioid antagonism is maximal at 24 hours in each case [4]. To achieve optimal selectivity, these compounds should therefore be administered at least 4 and preferably 24 hours before testing [4], [14], [15]. With an adequate pre-administration interval, none of the non-opioid activities we report here are likely to be detectable *in vivo*.

### Is JDTic a negative allosteric modulator of NOP?

Surmountable but noncompetitive antagonism of the kind exhibited by JDTic at NOP suggests allosteric modulation. However, it seems unlikely that JDTic binds to an allosteric site. JDTic protrudes deeply into the orthosteric site of κ-OR in the recently reported crystal structure [55]. C-24, a peptide mimetic derived from nociceptin, adopts the same pose in the orthosteric site of NOP, superimposable upon JDTic [56]. The binding pockets of κ-OR and NOP are extremely similar: dynA shows nanomolar affinity for NOP, and subnanomolar affinity for point mutants [29]. Therefore, the most plausible binding site for JDTic in NOP is the common orthosteric site exhibited in the crystal structures. This is puzzling given the apparent allosteric modulation we observed. Note, however, that allosteric modulation can occur between two orthosteric ligands bound to a receptor dimer [57].

## Conclusions

Our results confirm that nor-BNI, GNTI and JDTic bind with high selectivity to the κ-OR, extending previous results for opioid receptors to a broad panel of 43 non-opioid receptors and transporters. There was no non-opioid target for which all three compounds shared detectable affinity, or for which any two shared sub-micromolar affinity, while affinity for κ-OR was low nanomolar. Of the other interactions detected, the only one likely to occur in the CNS at typical concentrations is allosteric enhancement of α_1A_-AR by GNTI. This may partially account for that compound's severe transient effects. However, due to the low brain uptake of these compounds, achievement of effective central concentrations requires high plasma levels, which may briefly influence low-affinity peripheral receptors. For instance, GNTI acted as a weak M_1_ antagonist. Nonetheless, the κ opioid antagonism these compounds induce persists for weeks or months after these transient effects dissipate. With an adequate pre-administration interval, our results confirm that nor-BNI, GNTI and JDTic are exquisitely selective tools for the study of κ-OR *in vivo*.

## Materials and Methods

### Compounds

**GNTI**•2HCl•1.5H_2_O: Tocris Bioscience, Ellisville MI (batches 4B/91591 and 4B/94133). **JDTic**•2HCl•H_2_O: F. Ivy Carroll, Research Triangle Institute, NC. **Naltrexone**•HCl•2H_2_O: Tocris Bioscience (batch 5B/93329). **Nor-BNI**•2HCl•H_2_O: Tocris Bioscience (batches 8A/90732 and 9A/93084). Other compounds were purchased from Sigma-Aldrich (Milan, Italy).

### Binding and functional assays

Inhibition of the noradrenaline transporter (NET) was determined using fluorescent dye uptake (Neurotransmitter Transporter Uptake Assay Kit R8174, Molecular Devices, Sunnyvale CA) [58]. NOP binding assays were performed against [^3^H]N/OFQ in membranes prepared from HEK293T cells transiently transfected with human NOP as described previously [56]. Cyclic AMP inhibition by N/OFQ was determined in the same cells using a GloSensor™ assay (Promega Corp., Madison WI) as described elsewhere [59]. Briefly, cells were preincubated with antagonist for 15 min, then agonist for 15 min, before addition of luciferin and isoproterenol. Luminescence was measured after a further 20 minutes. Other functional and radioligand displacement assays were performed using standard PDSP protocols as described previously [60]. For all binding and functional assays, a minimum of three experiments were conducted in triplicate. For details of specific binding assays, see Table S1. Results were analyzed using nonlinear regression with Graphpad Prism 6.

### Caco-2 cell monolayer permeation

Caco-2 cells were grown in Dulbecco's Modified Eagle Medium (DMEM) with 10% heat-inactivated fetal calf serum, 100 U/mL penicillin, 100 µg/mL streptomycin, and 2 mM L-glutamine. The cells were trypsinized twice a week with trypsin/ethylenediaminetetraacetic acid (EDTA) (0.02% each) and the medium was changed twice a week. Cells were harvested with trypsin-EDTA and seeded onto a MultiScreen Caco-2 assay system (Millipore, Billerica, MA) at a density of 10,000 cells per well. The culture medium was replaced every 48 h for the first 6 days and every 24 h thereafter, and after 21 days in culture, the Caco-2 monolayer was utilized for the permeability experiments. Trans-epithelial electrical resistance (TEER) of the monolayers was measured daily before and after the experiment using an epithelial volt-ohm meter (Millicell-ERS; Millipore, Billerica, MA). TEER values were greater than 1800 Ω for 21-day cultures.

Apical to basolateral (P_app_, A→B) and basolateral to apical (P_app_, B→A) permeability of compounds were measured at 120 min and at various compound concentrations (1–100 µM). Compounds were dissolved in Hanks' balanced salt solution (HBSS, pH 7.4) and sterile filtered. After 21 days of cell growth, the medium was removed from filter wells and from the receiver plate. The filter wells were filled with 75 µL of fresh HBSS buffer and the receiver plate with 250 µL per well of the same buffer. This procedure was repeated twice, and the plates were incubated at 37°C for 30 min. After incubation time, the HBSS buffer was removed and compound solutions added to the filter well (75 µL). HBSS without the compound was added to the receiver plate (250 µL). The plates were incubated at 37°C for 120 min. After incubation time, samples were removed from the apical (filter well) and basolateral (receiver plate) side of the monolayer and then were stored in a freezer (−20°C) pending analysis. Concentrations were calculated from UV absorbance at the following wavelengths (nm): 278 (nor-BNI); 285 (GNTI); 276 (JDTic); 228 (naltrexone). The apparent permeability (P_app_), in nm sec^−1^, was calculated using the following equation:
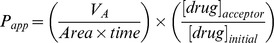
where V_A_ is the volume (in mL) in the acceptor well; Area is the surface area of the membrane (0.11 cm^2^ of the well); time is the total transport time in seconds (7200 s); [compound]_acceptor_ is the concentration of the compound measured by UV spectroscopy; [compound]_initial_ is the initial compound concentration in the apical or basolateral wells.

## Supporting Information

Dataset S1 **Raw data for** **Figure 2A** **.**
(XML)

Dataset S2 **Raw data for** **Figure 2B** **.**
(XML)

Dataset S3 **Raw data for** **Figure 3A** **.**
(XML)

Dataset S4 **Raw data for** **Figure 3B** **.**
(XML)

Dataset S5 **Raw data for** **Figure 4** **.**
(XML)

File S1 **Radioligand displacement curves.**
(PDF)

Table S1 **Binding affinities with standard error, radioligand, membrane type, and species.**
(XLS)
