# Supplementary material for: Selective κ Opioid Antagonists nor-BNI, GNTI and JDTic Have Low Affinities for Non-Opioid Receptors and Transporters
Source: PLoS One. 2013 Aug 14;8(8):e70701. doi: 10.1371/journal.pone.0070701 (PMC3747596; doi:10.1371/journal.pone.0070701)

Nor-BNI binding curves

(PDSP compound 14675)

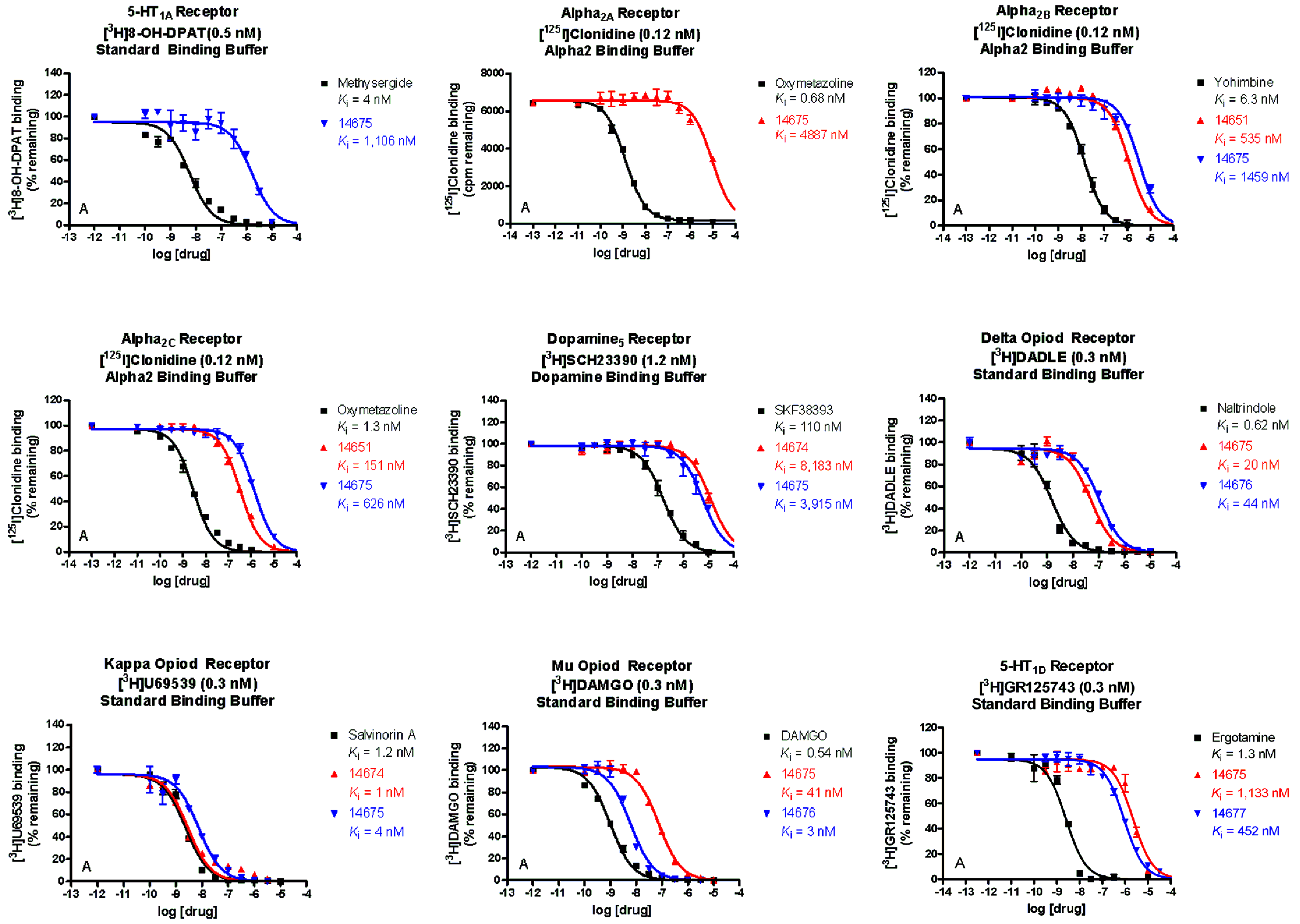

GNTI binding curves

(PDSP compound 14674)

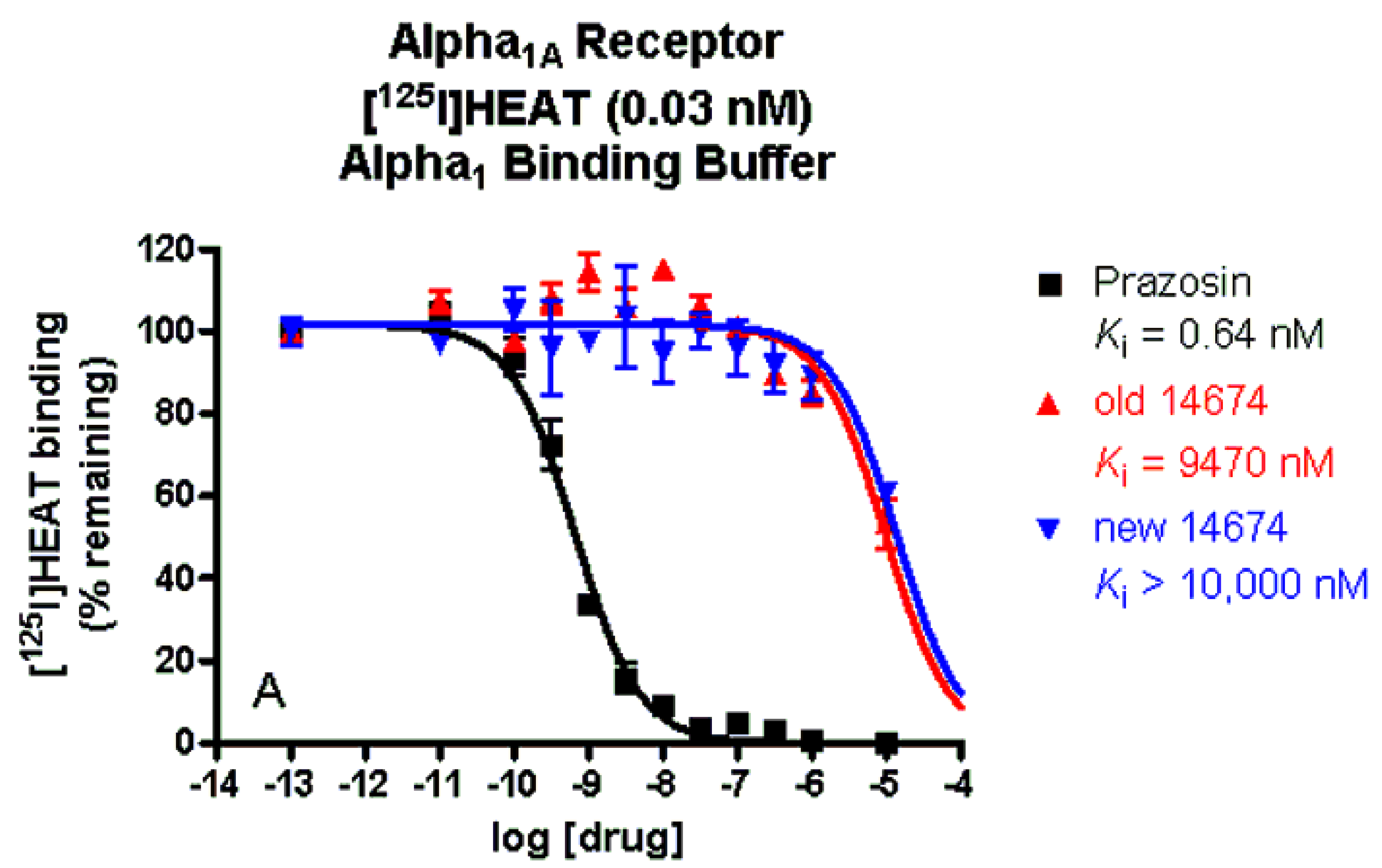

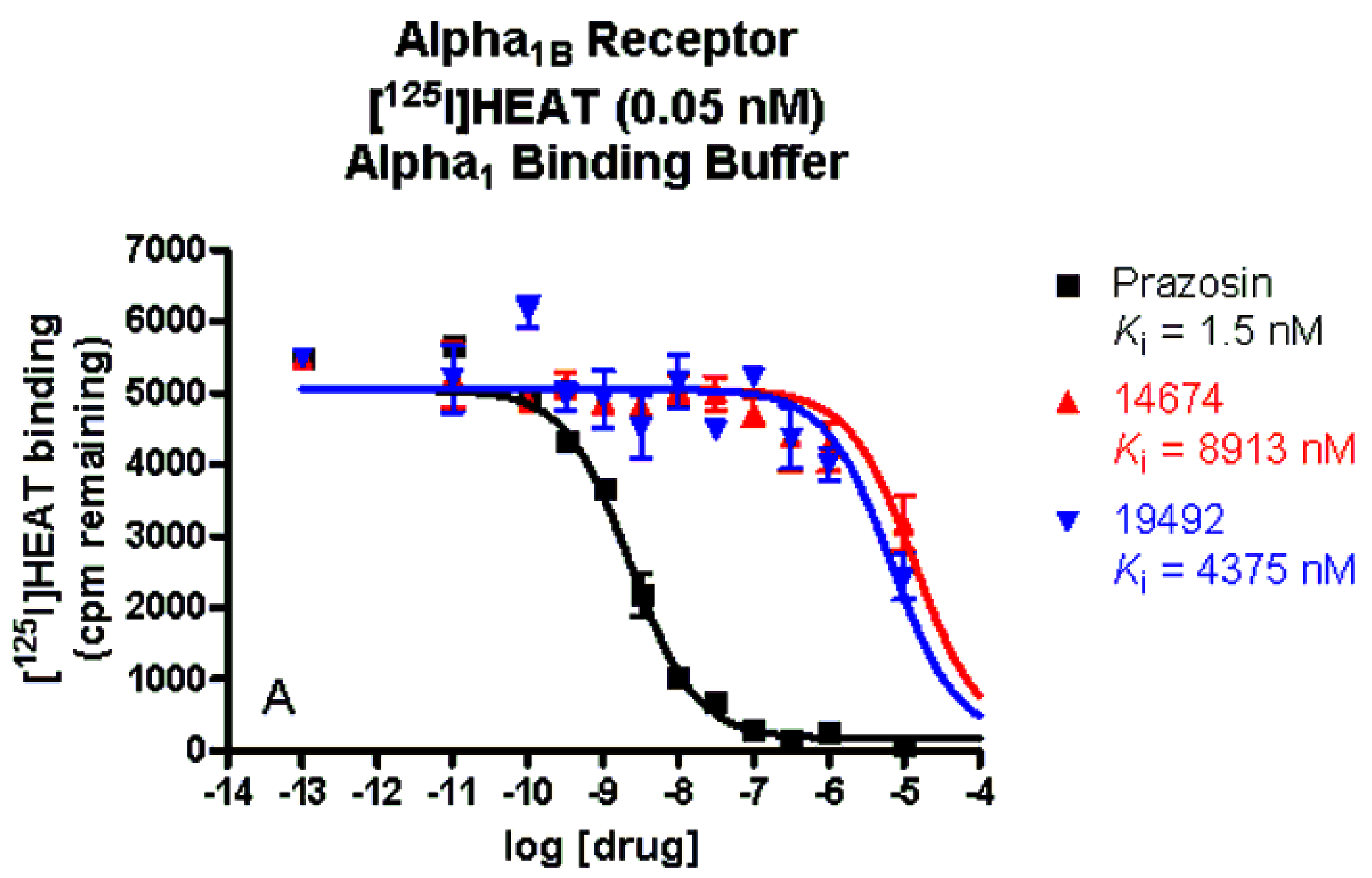

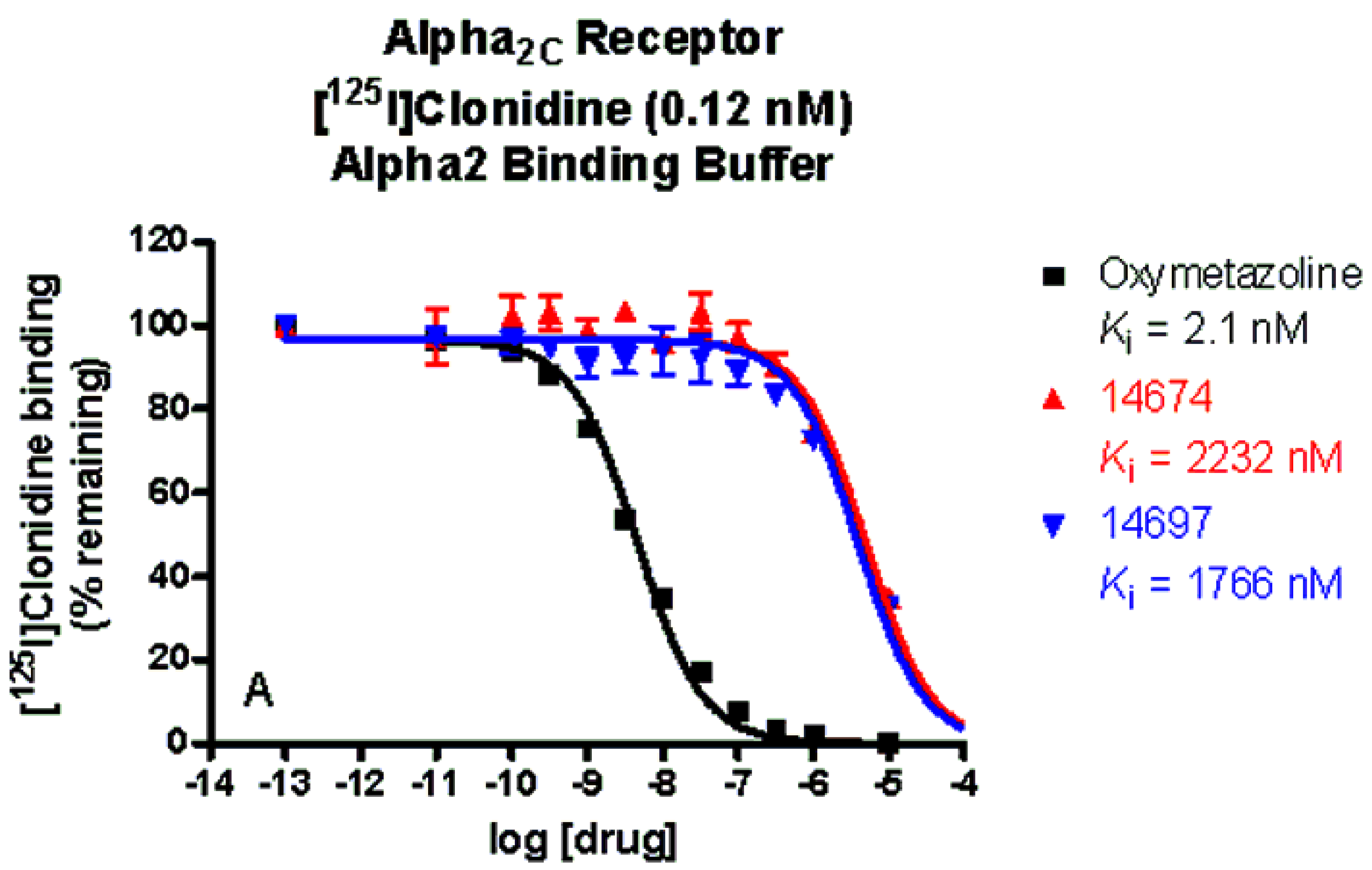

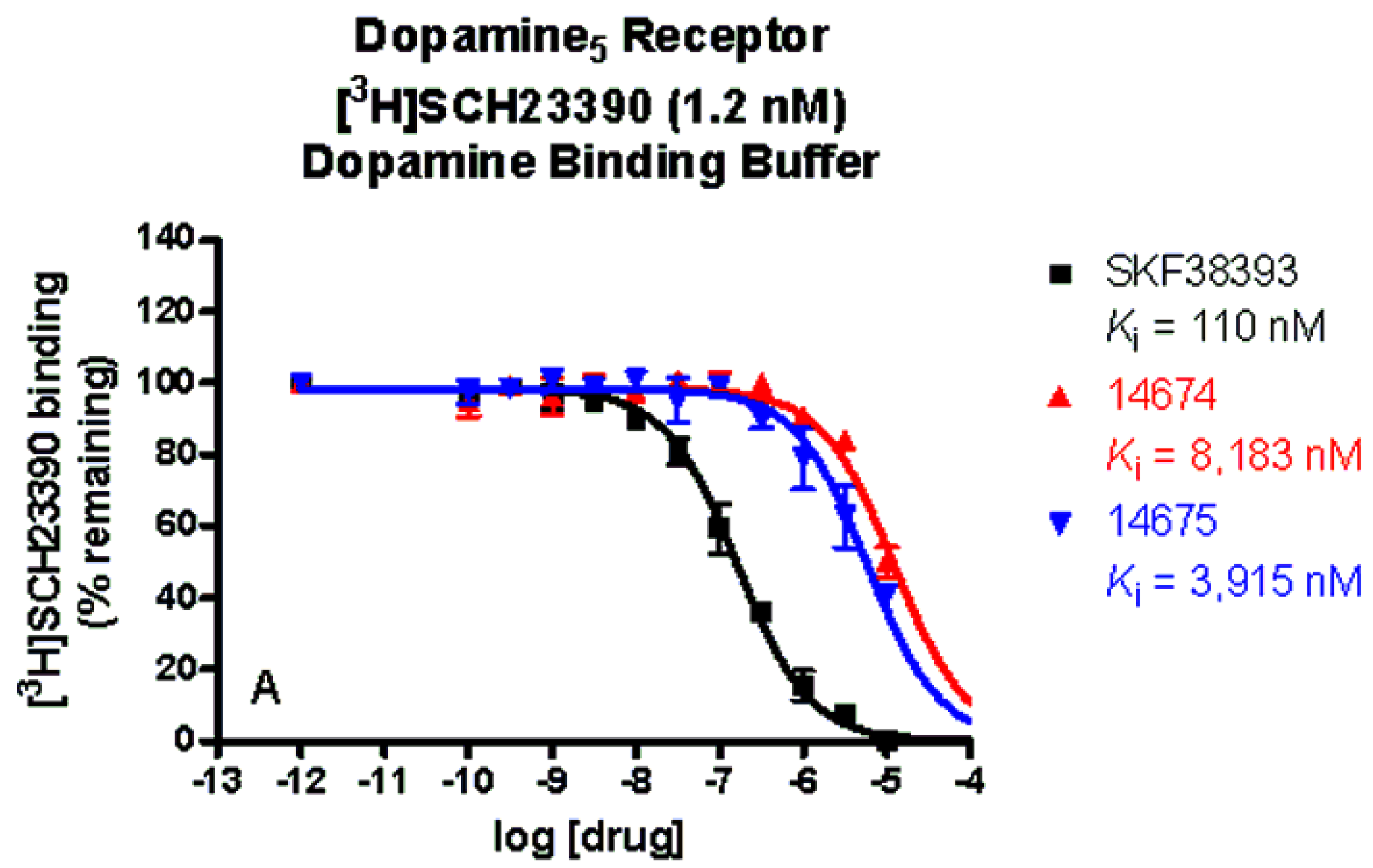

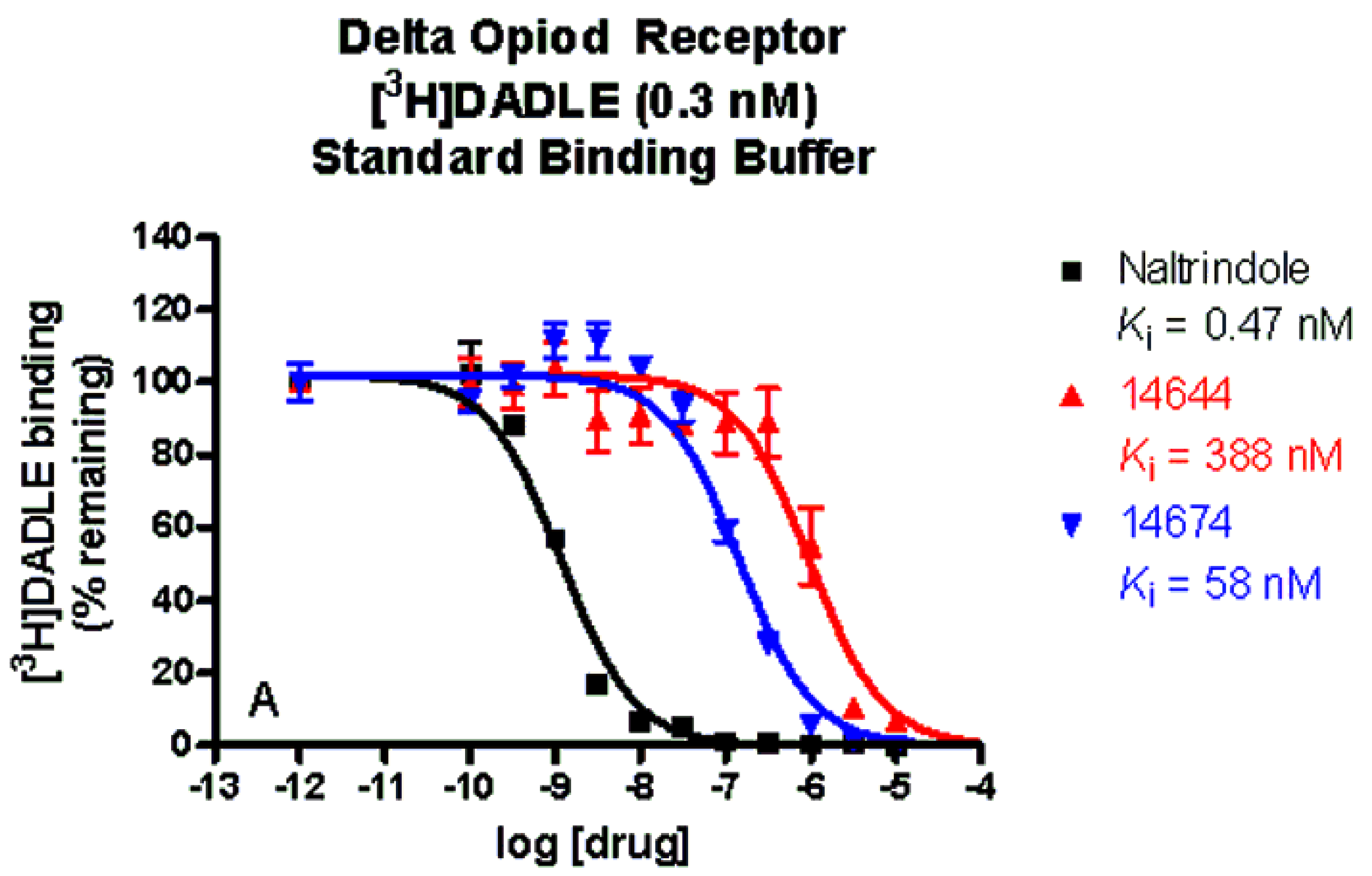

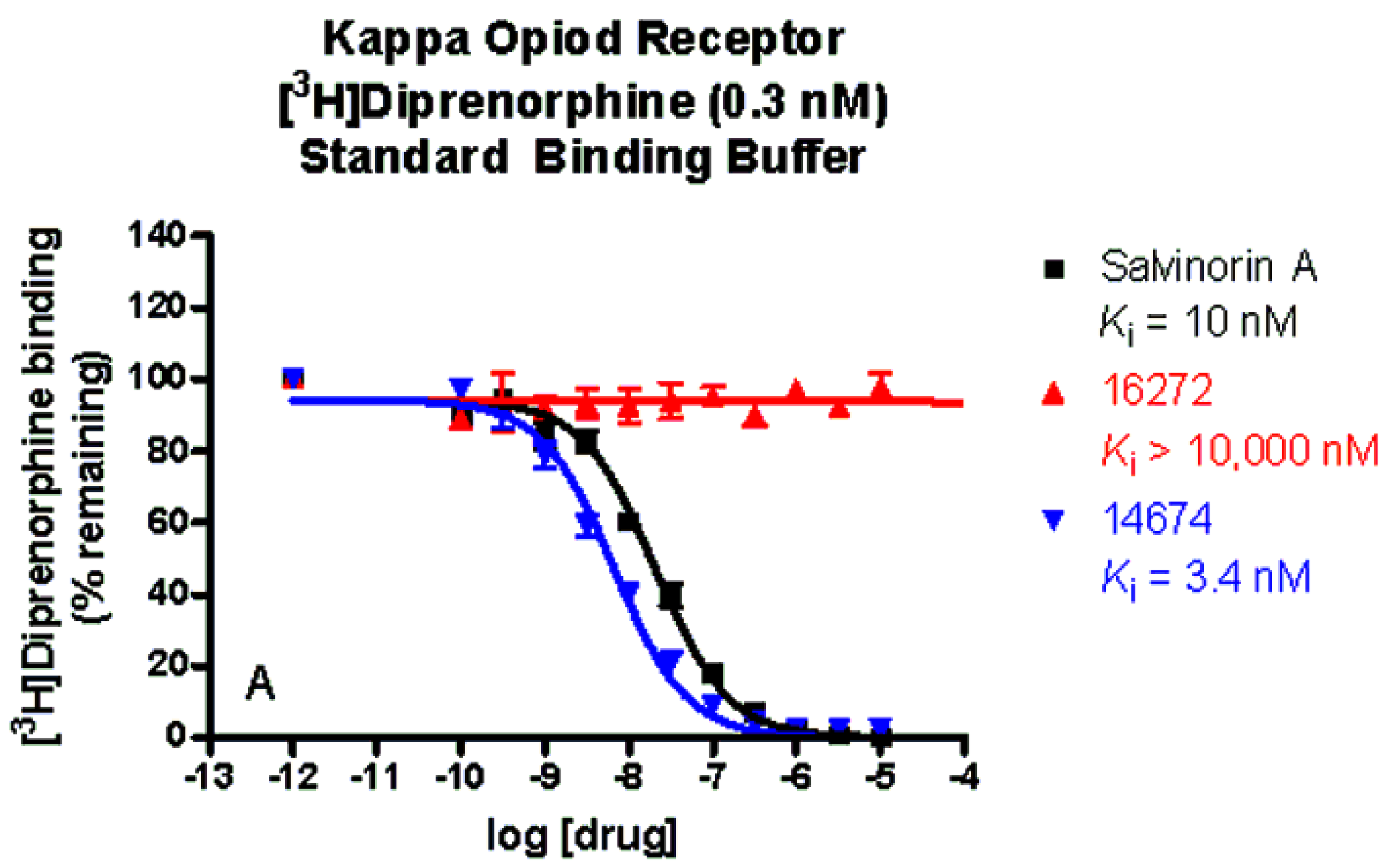

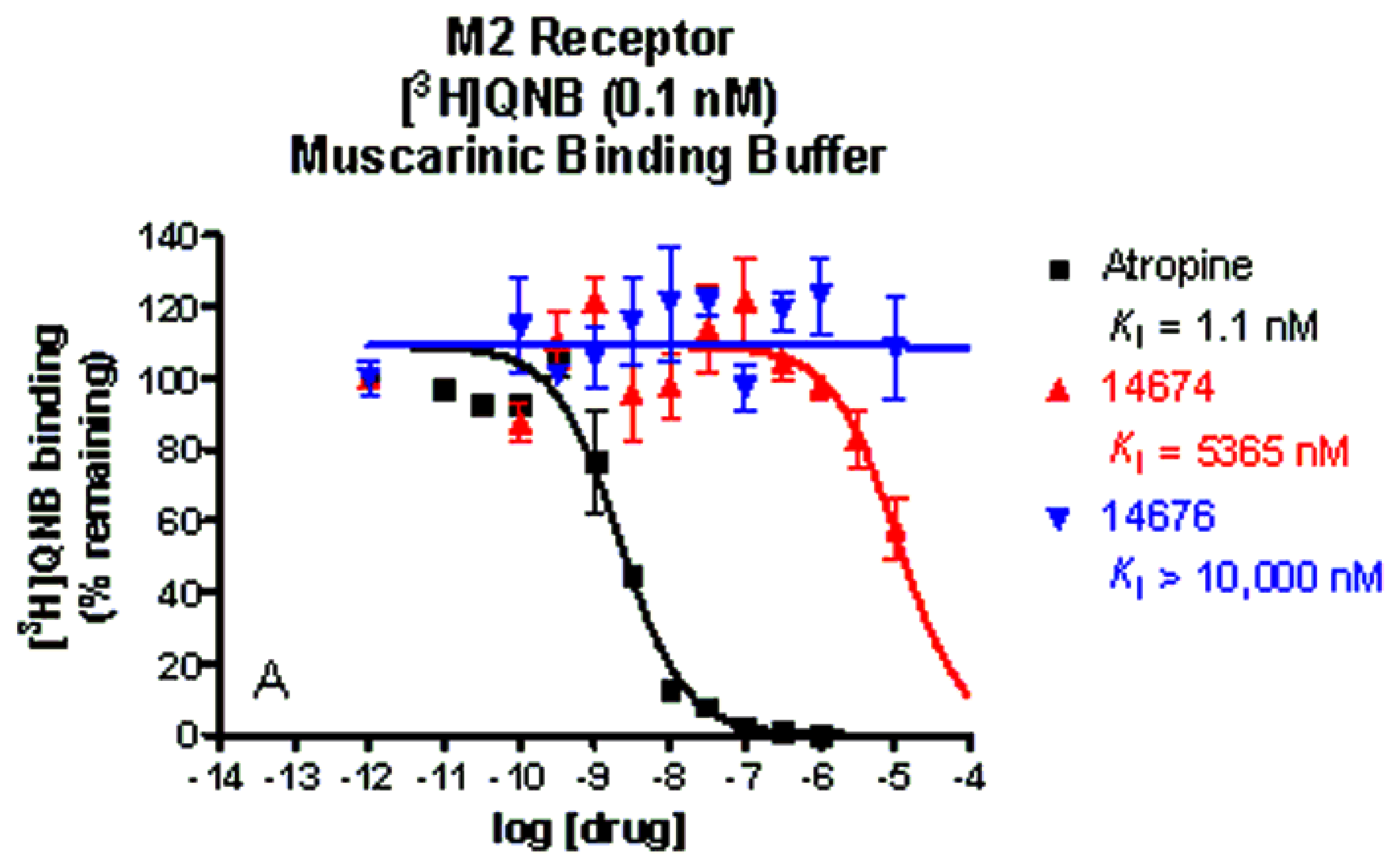

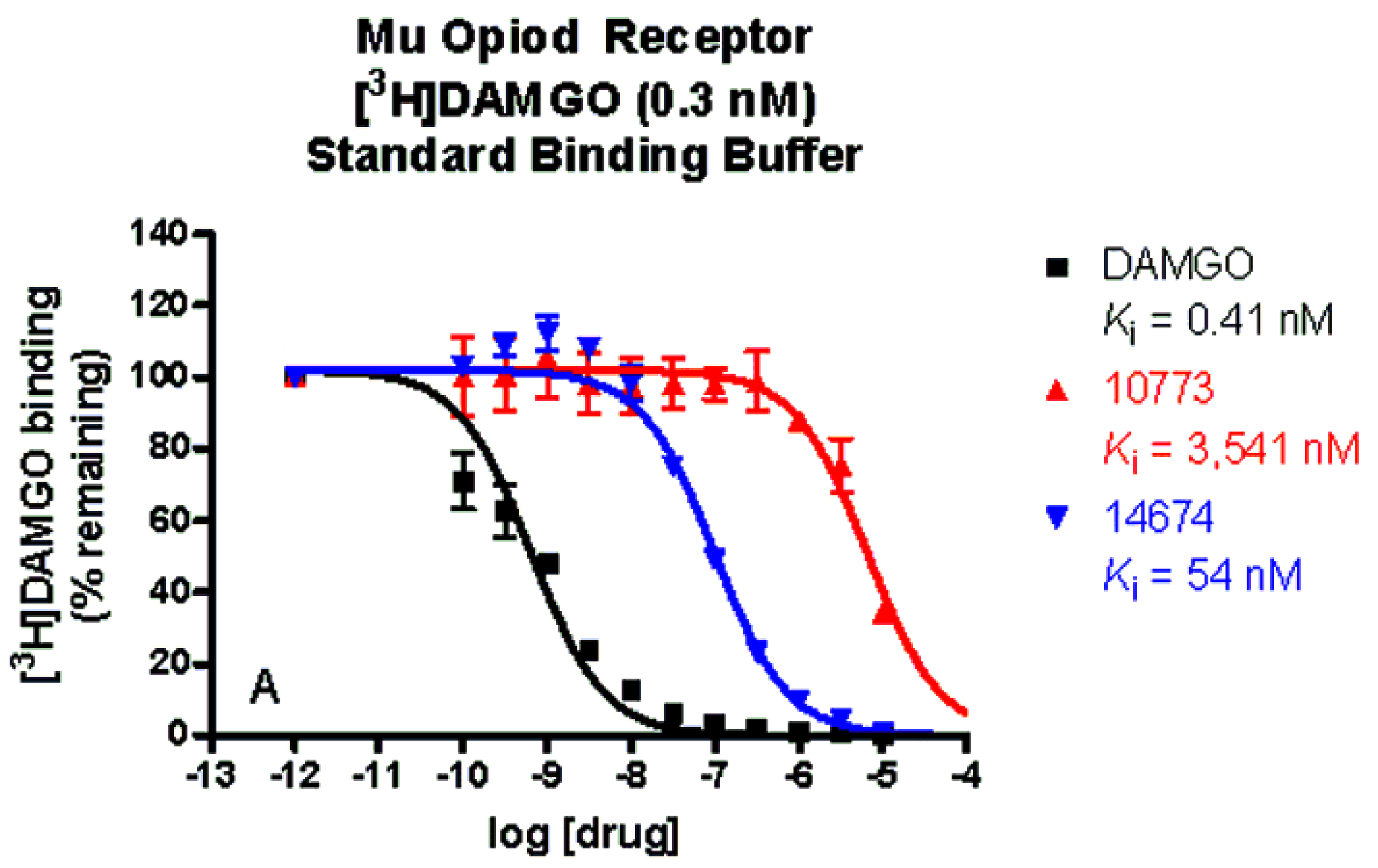

JDTic binding curves
(PDSP compound 14676)

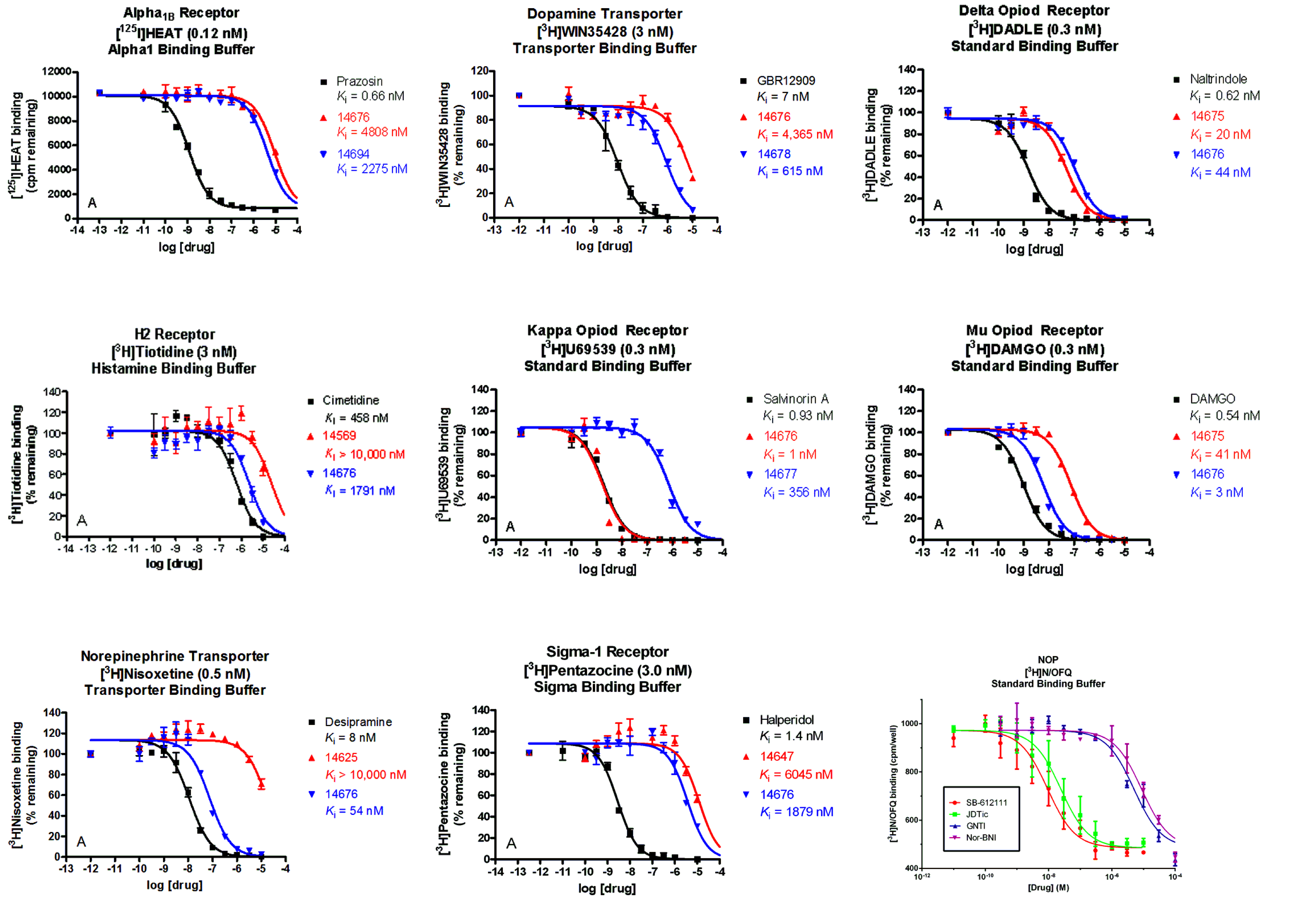

Supplement: File S1 — Radioligand displacement curves. (PDF) [file pone.0070701.s006.pdf]
